# Failed Early Intervention of Pyomyositis in an Immunocompetent Individual

**DOI:** 10.1155/2018/4296976

**Published:** 2018-11-22

**Authors:** Ranna Al-Dossari, Sana Zekri

**Affiliations:** ^1^University of North Texas Health Science Center, Texas College of Osteopathic Medicine, Fort Worth, TX, USA; ^2^John Peter Smith Health System, Fort Worth, TX, USA

## Abstract

Pyomyositis is a purulent infection of striated muscle tissue that usually leads to an abscess, commonly due to *S. aureus*. Pyomyositis is typically found in tropic regions, but it is increasingly being recognized in temperate climates, especially in immunocompromised individuals. Patient presentation ranges from afebrile with mildly elevated WBC to frank sepsis. In many reported cases, patients may develop multiple abscesses at different sites. We report a case of a 54-year-old male with a history of chronic obstructive pulmonary disease (COPD) presenting with right pectoral infection. This case demonstrates the possibility that antibiotic therapy in early presentations may not effectively prevent abscess formation, contrary to treatment suggestions found in the literature.

## 1. Introduction

The first reported case of pyomyositis in temperate climates was in 1971 [1]. Further reported cases demonstrated a predilection in adults with a 3 : 1 male-to-female ratio [1]. The pathophysiology of pyomyositis is unknown but is proposed to be due to hematogenous bacterial spread and seeding in muscle bodies of large skeletal muscles. Pyomyositis cases in tropic regions tend to occur in immunocompetent patients, in contrast to temperate disease presentations, which tend to occur in the immunocompromised individuals [2]. Patients may present with symptoms similar to those seen in necrotizing fasciitis and cellulitis, such as fever, pain, erythema, edema, and induration localized to a particular region [3], thus making diagnosis difficult. Septic shock is a common late-stage presentation [2]. The mortality rate associated with pyomyositis has been estimated to range from 1% to 20% [4].

## 2. Case Presentation

A 54-year-old Caucasian male with a past medical history of treated rhabdomyolysis, COPD, and occasional methamphetamine use presented to the emergency department for severe right pectoral pain that began 5 days prior. He reported coming to the emergency department 3 days prior to presentation with right pectoral pain and was put on sulfamethoxazole/trimethoprim for suspected cellulitis, but reported worsening of symptoms. Pain radiated from his right pectoral muscle to his right shoulder. He characterized the pain as severe, constant, sharp, and pleuritic.

He reported minor superficial abrasions from steel to his right superior lateral chest while working odd jobs one week prior to onset of symptoms, but denied any blunt trauma, intravenous drug use, recent travel, sick contacts, weight loss, or shortness of breath. He endorsed a fever the night prior to admission, nonproductive chronic cough, and red, swollen skin changes on his right chest.

Upon initial presentation, the patient was febrile and tachycardic, but stable. Patient's right chest was erythematous and tender to palpation along the distal right pectoral insertion and right shoulder, with 4/5 right shoulder strength and sensitivity to light touch along the right arm. His right chest was 3 times the size of his left chest. Nontender induration of the right flank was present. No discrete mass or abscess was palpable; however, a fluid collection was present in the posterior scapula region on the right. Lab results showed elevated levels of procalcitonin, c-reactive protein, and white blood cell count. The creatinine kinase level was not suspicious for rhabdomyolysis.

**Figure 1:**
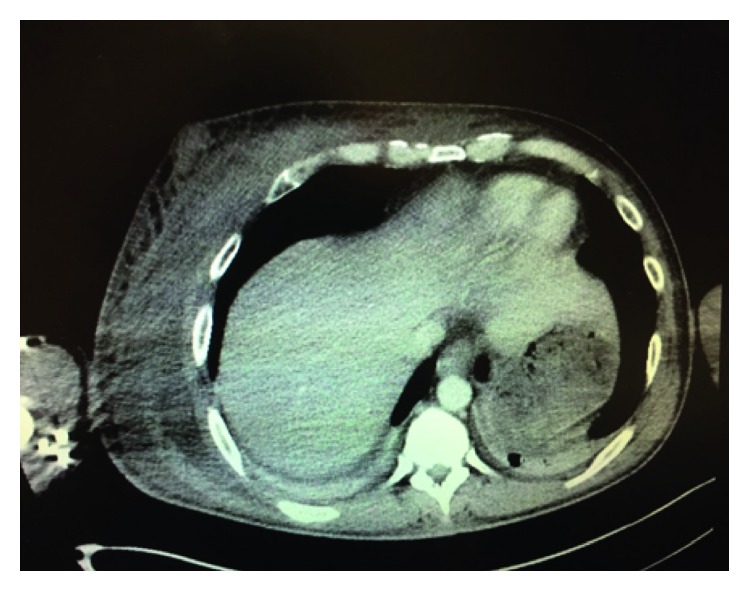
CT scan of chest with contrast at the level of the pectoralis muscle.

Chest x-ray was unremarkable. Initial ultrasound of his right pectoral muscle showed an area of skin induration with tiny anechoic areas compatible with cellulitis. Computed tomography (CT) scan of his chest showed fat stranding of the right pectoral, but no abscess (Figure 1).

Upon admission, the patient was placed on piperacillin/tazobactam and vancomycin. However, after ten days on the new antibiotic regimen, his clinical presentation failed to improve. Blood cultures were positive for *S. aureus* that was susceptible to vancomycin. Repeat ultrasonography was performed and showed interval development of abscess in the pectoral muscle body. Surgery was consulted, and they proceeded with incision and drainage. Surgical exploration revealed a large amount of purulent fluid in between the pectoralis minor and major muscles, and the diagnosis of pyomyositis was reached. Wound cultures were consistent with methicillin-resistant *S. aureus* (MRSA). The patient had improvement with serial drainages. In preparation for discharge, the patient's antibiotic regimen was switched to clindamycin for MRSA coverage and a nonparenteral route for administration. The patient was discharged in stable condition on oral clindamycin and a wound vacuum.

## 3. Literature Review

Temperate adult pyomyositis has an increased incidence in immunocompromised individuals. Immunocompromised states and common comorbidities include those with human immunodeficiency virus (HIV), diabetes mellitus, malignancy, cirrhosis, renal insufficiency, and use of immunosuppressive drugs. Other predisposing risk factors include trauma, IV drug abuse, and concurrent infections [5]. Pyomyositis cases in the United States have been commonly reported in patients with HIV infections, which may be linked to increased colonization of *S. aureus* in that population [3]. Furthermore, HIV patients taking antiretroviral therapy can have myopathies due to medication side effects, which renders the muscles prone to infection [3]. Additionally, trauma is an important predisposing factor, as it is reported in about 25–50% of pyomyositis cases [6]. Trauma leads to more iron flow in the muscle bed, providing a ripe environment for bacterial growth [6]. For example, athletes who perform vigorous activity develop minor muscle damage, suggesting that the pathophysiology of the infection in muscle tissue seen in pyomyositis may be due to hematoma formation or increased muscle perfusion from trauma [6].

The clinical course of pyomyositis can be broken down into three stages [7]. Stage 1 is considered the “invasive stage,” consisting of local myalgia, low-grade fever, pain, and edema. A deep abscess may not be palpable, but the area may be described as “rubbery quality” [2]. No purulent material will be present when attempting to aspirate muscle. Stage 2 is referred to as the “purulent stage” [2], occurring 10 to 21 days after the initial onset of symptoms and patients present with fever, severe muscle tenderness, and increased edema. A frank abscess may be clinically present [4]. Ninety percent of patients present to the hospital with stage 2 pyomyositis [7]. Stage 3 is a state of systemic toxicity with complications of *S. aureus* bacteremia [4]. Staging is important to delineate in assessing pyomyositis in order to choose the appropriate treatment. As mentioned above, early clinical stages may present similarly to necrotizing fasciitis and may be difficult to distinguish. Necrotizing fasciitis is a rapidly progressing infection that is often fulminant with a mortality rate of 25% to 75% [8]. It involves the superficial and deep fascia with necrosis and fluid collections [9]. Pyomyositis and necrotizing fasciitis may even coexist in tropical climates and in immunocompromised individuals in temperate regions [9]. Although swelling, erythema, and disproportionately severe pain can also be present in necrotizing fasciitis, the presence of blisters, bullae, and skin crepitus is strongly an indicative of necrotizing fasciitis [10].

Ultrasound and CT scan of the symptomatic region are often the first radiographic techniques used to evaluate for abscess formation, as plain radiographs are usually normal [8]. CT scans can be used to visualize diffuse muscle enlargement with heterogeneous attenuation on imaging due to fluid collections. Most commonly in pyomyositis cases, CT scans tend to show muscle enlargement with heterogeneous attenuation and focal rim enhancement in the inflamed muscle region [11]. Ultrasound is useful for image-guided abscess aspiration during the suppurative phase [8]. MRI is the most sensitive imaging modality to assess muscle inflammation, even in the early stages of pyomyositis before a frank abscess forms [9]. T2-weighted MRI often shows diffuse muscle enlargement and intramuscular abscesses [2]. MRI can also be used to distinguish pyomyositis and necrotizing fasciitis [2]. MRI can reveal areas of inflammation along the fascial plains and distinguish spread of infection into compartments and surrounding soft tissue structures [8].

Treatment for pyomyositis is based on staging. Stage 1 pyomyositis is treated with antibiotics in the hopes of preventing abscess formation. Stages 2 and 3 pyomyositis require percutaneous drainage or surgery of abscesses, as well as antibiotics. Antibiotics typically used include those with gram-positive and gram-negative bacteria coverage empirically, with narrowing of spectrum as appropriate [12]. In addition, anaerobic coverage should also be added early in treatment for immunocompromised patients [13]. Repeat drainage is commonly required. Antibiotics should be continued until clinical and radiographic improvement is confirmed [12]. Typically, patients recover after three to four weeks of parenteral antibiotic regimens. However, patients with poorly drained or multiple infections may require a longer duration antibiotic regimen [2].

## 4. Conclusion

The present case highlights the difficulty of detecting pyomyositis in its early stages without strong clinical suspicion and also points to a possibility that broad-spectrum antibiotics may not effectively treat stage I pyomyositis before abscess formation is achieved. The patient in the case report presented clinically in stage 1 pyomyositis. Surgical intervention is not recommended in the early stages of disease. As in the presented case, the recommended therapy of broad-spectrum antibiotics did not prevent abscess formation at the primary site. The role of antibiotics in early tropical pyomyositis may be to prevent continued hematogenous spread and subsequent appearance of further lesions; however, anecdotal experience from expert tropical medicine physicians indicates that antibiotics often do not prevent the formation of further abscesses. Surgery may be indicated in most cases at the primary site of infection with eventual abscess formation, regardless of initial antibiotic use. Although pyomyositis is relatively rare in temperate climates, this case demonstrates the importance of awareness of this tropical disease. In this case, the patient did not present as immunocompromised, which further adds to the difficulty of detecting the disease without high suspicion.
